# Truebeam Radiosurgery for the Treatment of Trigeminal Neuralgia: Preliminary Results at a Single Institution

**DOI:** 10.7759/cureus.1362

**Published:** 2017-06-16

**Authors:** Mena G Kerolus, Neilayan Sen, Sonal Mayekar, Alistar Templeton, Julius Turian, Aidnag Diaz, Lorenzo Munoz, Richard W Byrne, Sepehr Sani

**Affiliations:** 1 Neurosurgery, Rush University Medical Center; 2 Radiation Oncology, Rush University Medical Center

**Keywords:** linear accelerator, radiosurgery, radiosurgical rhizotomy, stereotactic radiosurgery, trigeminal neuralgia, truebeam, cyberknife, gamma knife

## Abstract

**Introduction:**

Radiosurgery is now an established method of satisfactory pain control in patients with trigeminal neuralgia (TN). The Varian Truebeam STx (Varian Medical Systems, Palo Alto, CA) linear accelerator (LINAC) system is an arc-based, frameless stereotactic radiosurgery system used for the treatment of TN. To our knowledge, there has been only one published series of patient histories that documents the use of a frameless LINAC system for the treatment of TN. We describe the treatment parameters, patient outcomes, and complications associated with the treatment of TN.

**Methods:**

All patients treated with the Truebeam system for TN between 2012 and 2015, with at least a six-month follow-up, were identified. A dose of 90 Gy was delivered to the isocenter using a 0.5 cm diameter cone. The cisternal segment of the trigeminal nerve was placed at the location of the LINAC isocenter using an ExacTrac™ (Brainlab, Munich, Germany) image guidance system. The radiosurgical dose, Barrow Neurologic Institute pain score (BNI PS), symptom recurrence, magnetic resonance imaging (MRI) radiographic changes, and other complications, including Barrow Neurologic Institute facial numbness score (BNI FN), were analyzed.

**Results:**

A total of 18 patients—15 women and 3 men—with a mean age of 58 years (median: 59 years; range: 22-84 years) were treated at our institution. Fourteen patients (78%) had a BNI PS of IIIb or better, which was considered successful treatment. Twelve patients had excellent (BNI PS I) pain relief and two patients had good (BNI PS II-IIIB; recurrence after one year) pain relief. The pain of four patients recurred after a mean of 10 months.

**Conclusion:**

Truebeam radiosurgery can provide effective and safe treatment for patients suffering from TN. The efficacy appears similar to other frame- and frameless-based systems

## Introduction

Trigeminal neuralgia (TN) affects up to 12 out of every 100,000 people. Various classifications have been proposed to describe TN and facial pain syndromes; Burchiel [1] created the most accepted classification scheme. TN types 1 and 2 are described as lancinating, paroxysmal, electric shock-like lasting pain that is either episodic as in TN type 1 or constant as in TN type 2. First-line treatment for TN involves medical therapy with medications such as anticonvulsants. Surgical options include microvascular decompression and ablative procedures, such as percutaneous balloon microcompression, radiofrequency rhizotomy, glycerol rhizolysis, and radiosurgery [2]. Since Leksell introduced radiosurgery as a treatment option for TN in 1951 [3], frame-based radiotherapy, such as Gamma Knife (Elekta AB, Stockholm, Sweden) surgery, has been established as an effective treatment option for pain relief in patients with TN [2,4,7].

Frameless radiosurgery has been used successfully as a method of treatment for TN [8-9]. CyberKnife (Accuray, Inc., Sunnyvale, CA) consists of a six-axis robotic arm that uses real-time image guidance with frameless positioning and a linear accelerator (LINAC)-based system to deliver radiation. Over the past decade, the use, accuracy, efficacy, safety, and success of frameless radiosurgery have been described with results comparable to frame-based systems.

The Varian Truebeam (Varian, Palo Alto, CA) system is a LINAC-based platform that has been used for the treatment of TN [9-10]. Chen et al. reported a 15-month follow-up of patients treated using a frameless LINAC system with patient outcomes similar to published data [9]. In early 2012, we began treating patients using the Varian Truebeam system and now report the clinical outcomes of 18 patients after a mean follow-up of 27.5 months 

## Materials and methods

### Patient selection

All data acquisition and analysis was approved by the Institutional Review Board. A total of 21 patients were treated with the Truebeam system for TN refractory to medical or surgical procedures. Three patients were lost to follow-up. Two of these patients had complete resolution of their symptoms at 56 and 99 days, respectively, based on the BNI PS. One patient noted that her pain was unchanged at her two-month follow-up. These patients were not included in our analysis. In our remaining cohort of 18 patients, clinical outcomes were assessed at a mean follow-up time of 27.5 months (median: 27.7 months; range: 6-40.7 months). Demographic and treatment information was obtained through a retrospective chart review. Post-treatment follow-up data was collected through outpatient patient charts or through phone interviews.

All patients underwent pretreatment evaluation, including comprehensive clinical history-taking, physical evaluation, and appropriate imaging studies, including a fast imaging employing steady-state acquisition (FIESTA) MRI scan. Patients who failed medical therapy, were ineligible, or declined surgery were offered radiosurgery. 

### Demographics 

A total of 18 patients—15 women (83%) and three men (17%)—were included in this study. Ten patients (56%) had TN type 1, four patients (22%) had TN type 2, and four patients (22%) had TN type 5 (history of multiple sclerosis (MS)). The average age at treatment was 56.3 years, with a median age of 58 years (range: 22-84 years). Five patients (28%) had undergone unsuccessful prior nonmedical intervention, which included microvascular decompression, radiofrequency or retrogasserian glycerol rhizotomy, and prior radiosurgery at a different institution. Pain distribution and selected demographic characteristics are presented in Table 1.

**Table 1:** Patient demographics V1 = ophthalmic division; V2 = maxillary division; V3 = mandibular division

Characteristic	N (%)
No of patients	18
Women	15 (83)
Men	3 (17)
Age (years) (mean (range))	56.3 (22-84)
Patients with prior surgery	5 (28)
TN Type 1	10(56)
TN Type 2	4 (22)
Multiple sclerosis	4 (22)
Laterality	
Left	8
Right	10
Distribution	
V1	0 (0)
V2	4 (22)
V3	5 (28)
V1 + V2	2 (11)
V2 + V3	6 (33)
V1 + V2 + V3	1 (6)
Average follow-up (months)	27.5

Treatment planning

An MRI was obtained at 2-21 days prior to treatment. Patients were immobilized with an S-frame mask (QFix, Avondale, PA) and thin slice (1.5 mm) axial computed tomography (CT) images were obtained at the time of simulation using helical acquisition by a 16-slice Philips Brilliance (Phillips Healthcare, Amsterdam, Netherlands) CT scanner. CT images were fused with FIESTA axial slices (0.8-mm slice thickness) for target delineation. The trigeminal nerve root was identified on the FIESTA MRI. The brainstem was contoured on consecutive axial slices on CT with the aid of MRI fusion. A five mm diameter BrainLab (Westchester, IL) cone was used for treatment in all cases. A total of seven 6 MV flattening filter-free noncoplanar arcs were designed to deliver 90 Gy to the isocenter of the cisternal segment of the trigeminal nerve root. The segment of the trigeminal nerve root treated was determined based on the dose isodose lines (IDL) which corresponded to the 50% IDL (45 Gy) abutting the brainstem. This corresponded roughly to the dorsal root entry zone, which is approximately 3 mm from the edge of the brainstem.

Prior to treatment, Winston-Lutz testing was performed to confirm radiation, mechanical, and imaging isocenter coincidence with a tolerance of 0.5 mm. All patients were treated in a single treatment session. 

### Treatment delivery

Patients were positioned supine on the treatment couch using a frameless thermoplastic mask. Stereoscopic orthogonal radiographic images using ExacTrac™ stationary x-ray tubes were obtained to verify the position with a tolerance of 0.75 mm and 1 degree of rotation. Six-degrees-of-freedom positional changes were applied based on these radiographs. A kV cone beam CT scan was performed to confirm the stereoscopic x-ray setup.

ExacTrac™ x-ray verification images were taken every 10-20 arc degrees during treatment delivery. If the verification alignment suggested a mismatch greater than 1 mm, treatment was interrupted and patient repositioning performed.

All patients received 10 mg dexamethasone prior to treatment and were discharged on the same day after treatment with a solumedrol dosepak. 

### Clinical and radiographic outcome evaluation and follow-up

Initial follow-up for all patients was done between three and six months after treatment. Patients were seen sooner if there were any concerns or relapse of pain. Follow-up data was collected after an initial visit through a phone interview or outpatient clinic documentation. Patients were evaluated for the level of pain control, response rate, time to pain relief, the occurrence of hypesthesia, and time to pain recurrence.

Post-radiosurgery pain was assessed using BNI PS, as shown in Table 2 [11]. The BNI FN was used to assess facial numbness, as shown in Table 3 [12].

**Table 2:** BNI PS

I	No pain and not on medication
II	Occasional pain but not requiring any pain medication
IIIa	No pain, with continued medication
IIIb	Some pain, controlled with medication
IV	Some pain, not controlled with medication
V	Severe pain and no pain relief with medication

**Table 3:** BNI FN

I No facial numbness
II Mild facial numbness, which is not bothersome
III Somewhat bothersome facial numbness
IV Very bothersome facial numbness

Successful treatment was defined as a BNI score of I-IIIb. A BNI score of I was deemed an excellent outcome, while a BNI score of II-IIIb and recurrence after one year was a good outcome. Treatment failure was defined as a BNI score of IV or V, with recurrence prior to one year. Recurrence was defined as a relapse to a prior lower level after a higher level was achieved after initial treatment (Table 4).

**Table 4:** Outcome measures using BNI PS

Successful	BNI Score I
Excellent	BNI Score I-IIIB
Good	BNI Score 1-IIIB, recurrence after 1 year
Poor	BNI Score IV, V, recurrence prior to 1 year

A post-radiosurgery MRI scan was obtained approximately two months after radiosurgery. An MRI was obtained sooner in situations of new neurologic changes or treatment failure. 

### Statistical methods

A Fischer’s exact test and univariate analysis were used to determine the correlation between patient co-morbidities and treatment success. A P value of <0.05 was used for statistical significance.

## Results

The median BNI PS was four prior to radiosurgery. Five (28%) patients had BNI PS III, nine (50%) patients had BNI PS IV, while four (22%) patients had BNI PS V.

Six patients (33%) had pain distribution involving both the maxillary (V2) and mandibular (V3) divisions. Five patients (28%) had V3 pain, four (22%) had V2, two (11%) had ophthalmic (V1) division and V2 pain, and one (6%) had pain in the V1, V2, and V3 distributions. 

### Pain relief

Patients were followed for an average of 27.5 months (median: 27.7 months; range: 6-40.7 months). At the 12-month follow-up, 17 patients (94%) reported some improvement in pain. At the last follow-up, 13 patients (72%) had successful pain relief (BNI PS I-IIIb). Eleven of these patients had excellent pain relief (BNI PS I) and two patients had good pain relief (BNI Score II-IIIB, with recurrence after one year). 

Symptom recurrence was noted in four patients. This occurred within one year and the cases were deemed treatment failures. The mean time to failure was 10 months. One patient had a preoperative BNI PS of III, two patients had a BNI PS of IV, and one patient had a BNI PS of V. One patient with BNI PS III and a history of MS was re-treated with radiosurgery at 240 days and remained pain free (BNI PS I) at his nine-month follow-up. The remaining three patients did not wish to undergo further radiosurgery.

Ten patients (56%) had TN type 1. Eight patients had excellent results, one patient had good results, and one patient had a recurrence of symptoms. Four patients had MS. Prior to treatment, two of these patients had a BNI PS of III and two patients had a BNI PS of IV. Three patients with MS had successful radiosurgical outcomes. One patient experienced a recurrence of his symptoms but after a second radiosurgery treatment, he has remained pain free. Three ultimately had excellent (BNI PS I) pain relief and one had good (BNI PS II) pain relief.

Five patients failed prior radiosurgical or surgical treatment for TN before undergoing radiation at our institution. Four patients had prior microvascular decompression (MVD) and one patient underwent Gamma Knife radiosurgery at another institution. Three of these patients had complete resolution of their pain after radiosurgery, with pain relief for a mean duration of 27.6 months (median: 27.7 months; range: 19-36 months).

**Figure 1:**
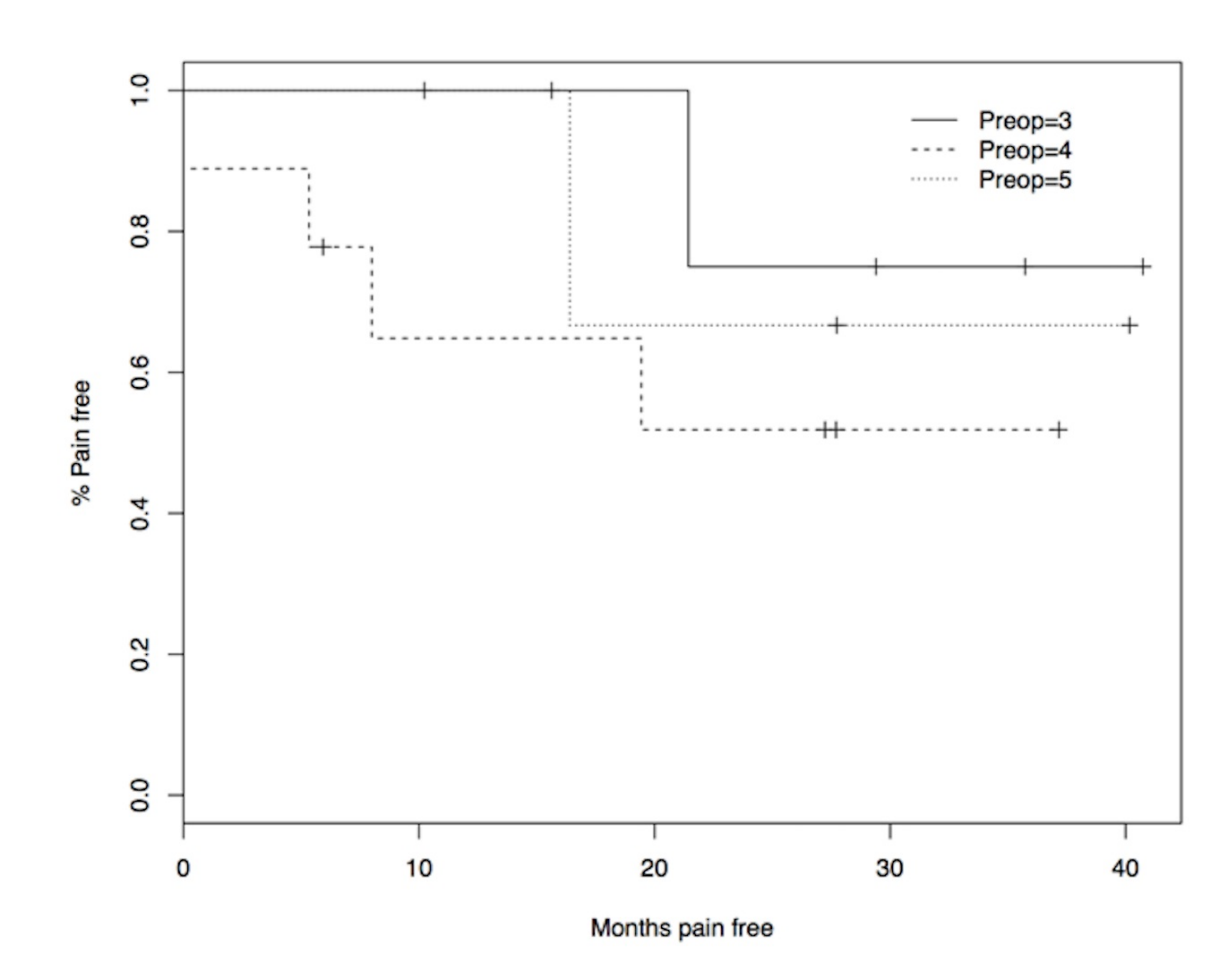
Pain-free survival based on patients’ preoperative BNI PS

There were four patients with a BNI PS of V prior to treatment, of which three had complete resolution of symptoms with an average follow-up of 27.8 months (median: 27.7 months; range: 15.6-40 months). One patient with a BNI PS of V had temporary pain relief for 16 months but the pain reoccurred. A pain-free survival curve based on preoperative BNI PS is shown in Figure 1.

The mean time to pain relief in all patients was 82 days (median: 40 days; range: 7-365 days). Patients with MS had a mean time to pain relief of 47.5 days (median: 36 days; range: 28-90 days). Patients with previous surgical intervention had a mean time to pain relief of 22 days (median: 21 days; range: 7-40 days). Overall, patients experienced an average of 22.5 months of pain relief (median of 24.3 months).

### Side effects

Seven patients (39%) had nonbothersome facial numbness (BNI FN II), and one patient had bothersome facial numbness with a persistent itching sensation in her eye (BNI FN III). Interestingly, none of the patients who underwent prior radiosurgery treatment (two patients) experienced facial numbness. One patient reported dry eye syndrome, which resolved after nine months.

### Dosimetry results

**Figure 2:**
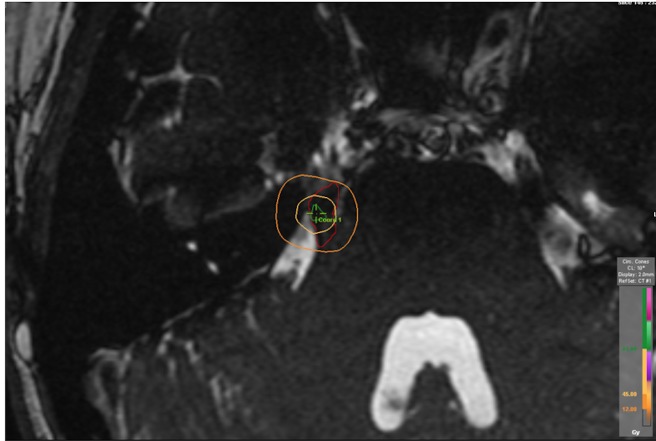
Treatment isodose distribution Treatment isodose distribution for a patient with right TN, overlaid on a volumetric T2 image; the red oval outlines the trigeminal nerve, the green contour represents the 90% isodose line (81 Gy), the inner orange circle is the 50% isodose line (45 Gy), and the outer orange circle outlines the 13.3% isodose line (12 Gy).

The average volume of the delineated trigeminal nerve was 0.069 cm^3^ (range: 0.036 cm^3^ – 0.15 cm^3^). The average volume of the trigeminal nerve encompassed by the 90% IDL (81 Gy), 50% IDL (45 Gy), and 13.3% IDL (12 Gy) were 0.014 cm^3^ (range: 0.007 cm^3^–0.022 cm^3^), 0.033 cm^3^ (range: 0.014 cm^3^–0.057 cm^3^), and 0.057 cm^3^ (range: 0.029 cm^3^–0.088 cm^3^), respectively. A typical isodose distribution is shown in Figure 2. The mean D50 (dose encompassing 50% of the contoured trigeminal nerve) was 46.2 Gy (range: 13.9 Gy – 72.5 Gy). The percentages of the trigeminal nerve treated at 90%, 50%, and 13.3% were 24%, 51%, and 85%, respectively. The average diameter of the 50% (45 Gy) IDL was 0.393 cm (range: 0.299 cm – 0.477 cm) and the spherical volume was 0.121 cm^3^ (range: 0.070 cm^3^ – 0.178 cm^3^) (Table 5 and Table 6).

**Table 5:** Dosimetry results

V_Total_ TN	Avg V_10%_	Avg V_50%_	Avg V_90%_	Avg D_50%_
(cc^3 ^)	(cc^3^ )	(cc^3^ )	(cc^3^ )	Gy
0.069	0.014	0.033	0.057	46.2

**Table 6:** Percent of trigeminal nerve treated

Avg _%_ of TN treated at 10% IDL	24%
Avg _%_ of TN treated at 50% IDL	51%
Avg _%_ of TN treated at 90% IDL	85%

### Post-treatment surveillance imaging

**Figure 3:**
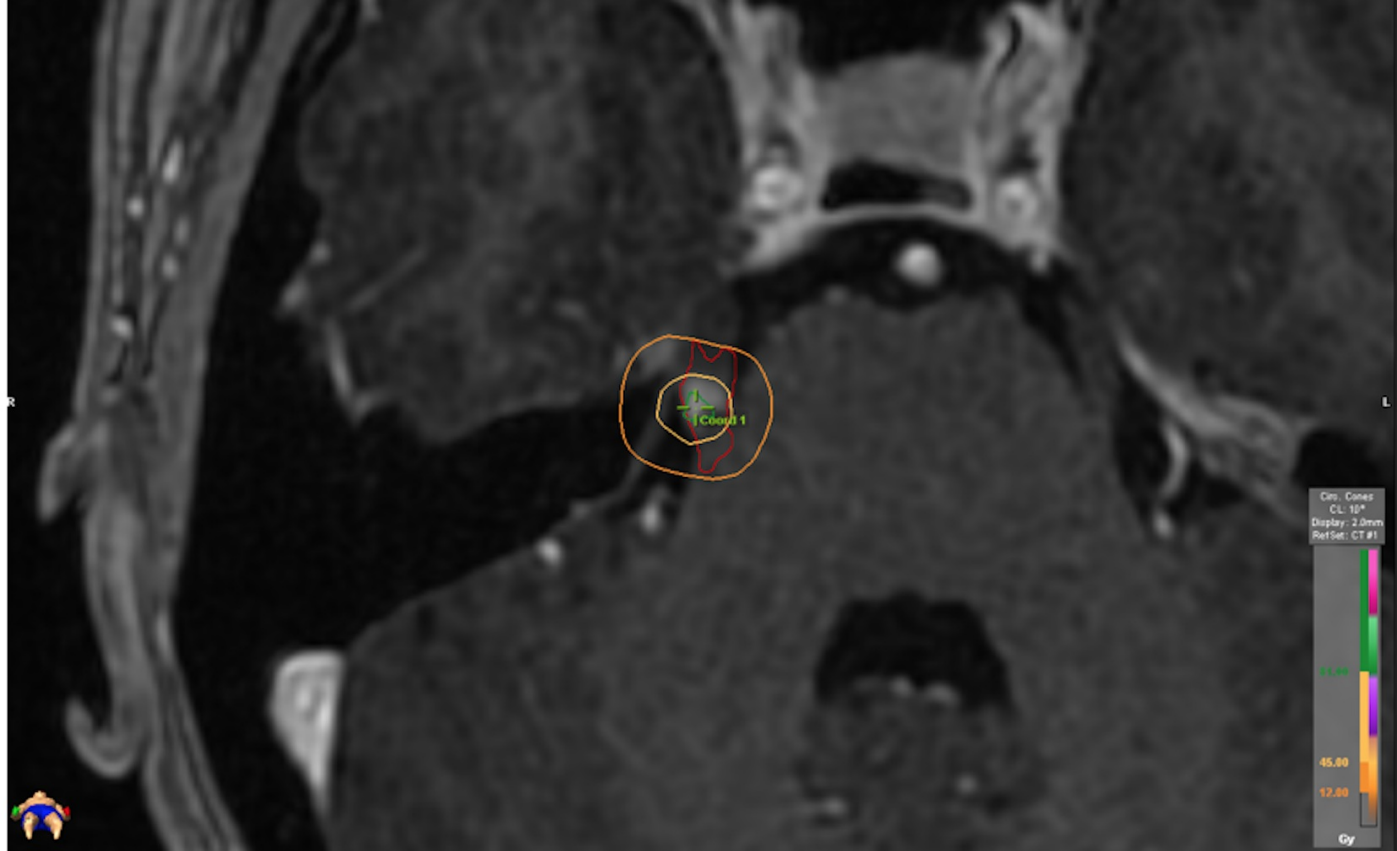
Isodose distribution with MRI changes The treatment isodose distribution of a patient with MRI changes at the root entry zone of the trigeminal nerve, depicted on a volumetric T1 image series with gadolinium contrast; note that the MRI changes remain within the 50% isodose line (45 Gy).

A post-treatment surveillance MRI was obtained in 17 patients at a mean of 78 days after treatment (median: 57 days; range: 12 – 257). Six patients (33%) had evidence of contrast enhancement within the treated volume (50% IDL), as seen in Figure 3. Four of these six patients experienced an excellent (BNI PS I) response to treatment, although the excellent outcomes did not statistically correlate to MRI changes. One patient with a history of MS had MRI changes outside of the treated volume and experienced an excellent (BNI PS I) response to treatment. MRI changes correlated with a median IDL of 41.0 Gy (range 2.3 Gy – 60.6 Gy, designated by the minimum peripheral dose of the identified radiologic changes).

## Discussion

Treatment modalities for TN consist of conservative medical therapy, surgical decompression, ablative procedures, and radiosurgery. Surgical intervention carries risks such as hemorrhage, infections, and cranial nerve palsies with morbidity rates of up to 3% [13-14]. These procedures provide pain control 60% to 100% of the time with a risk of sensory deficit of up to 3%-55% [15]. Surgical intervention, such as microvascular decompression, remains an excellent choice for treatment, given its early pain relief response, longevity of pain relief, and durability [15]. However, not all patients are candidates for surgery, given comorbidities or personal preferences, and radiosurgery is an acceptable and safe treatment option for TN.

The configurations of frame-based systems, including Gamma Knife, use rigid fixation of the head and have established Gamma Knife as a safe and efficient treatment option for TN [6]. The overall treatment success using Gamma Knife ranges from 85%-96% [4,6]. Ninety percent of patients will have pain relief within 6-12 months after the procedure with Gamma Knife, and the durability of treatment ranges from 40%-94% for two to five years after treatment [5,7,16].

CyberKnife and select LINAC-based systems can use frameless-based positioning for the accurate delivery of radiation. Using the ExacTrac™ x-ray system, head position monitoring can be performed on-demand during radiation delivery. We found that frameless image-guided radiosurgery provides convenience when acquiring imaging and a shorter set-up time on the day of radiation. It is also painless, which is similar to other published studies [9].

Radiosurgery involves a focused beam arrangement directed to the trigeminal nerve at the root entry zone at the anterolateral portions of the pons. The isocenter receives the maximum point dose, which is most frequently advocated for at the dorsal root entry zone, approximately 2-3 mm from the edge of the brainstem. The root entry zone is advocated as the ideal location of treatment, given the histologic changes in the nerve from the compact axonal fibers and oligodendrocytes layers of myelin in the central nervous system to the Schwann cells of the peripheral nervous system [16]. Other treatment targets include the gasserian ganglion or the cisternal nerve proximal to the gasserian ganglion; however, these results are mixed [15]. Our treatment planning involved contouring the radiation dose so that the 50% IDL (45 Gy) was abutting the brainstem, which corresponded to the dorsal root entry zone 3 mm from the brainstem. Contouring of the IDL is unique to LINAC-based systems over Gamma Knife. The dorsal nerve root entry zone was advocated, given successful results in previously published studies.

Given the proximity to the brainstem and other critical neural structures, validation was an important step toward the acceptance of frameless radiosurgery as an accurate method for intracranial radiosurgery. The targeting error during frameless radiosurgery is comparable to frame-based treatment methods, with reports ranging from a 0.26-0.32 mm error in translation [17-18].

Various nerve lengths, volumes, and dosing in the treatment of TN have been described in the literature. An increase in length or volume has not been correlated to better outcomes. With Gamma Knife, longer portions of the nerve were treated using larger collimator sizes with the hope of increased efficacy but this only caused a higher incidence of facial numbness [19]. Similarly, when using CyberKnife, Chen et al. found that the treatment of longer nerves and higher doses of radiation were only associated with a minimal increase in pain relief, with a substantially higher risk of hypoesthesia [20-21]. The volume of nerve treated using LINAC-based systems has also shown similar results. Goss et al. published the first series on LINAC-based treatment of TN in 25 patients with a treatment of 90 Gy using the frame-based Novalis (Brainlab, Westchester, IL USA) system. A 5-mm collimator to deliver 90 Gy to the nerve entry zone with a 50% IDL at the margin of the brainstem was employed. The correlation of facial numbness and the mean dose of brainstem exposed was statistically significant [22]. In our study, the 50% IDL (45 Gy) was placed adjacent to the brainstem, treating 51% of the volume of the TN. Since we were able to obtain the volume of the cisternal segment of the trigeminal nerve treated, we found that the smaller fractional volume of nerve treated at the 45 Gy IDL (or higher) had a worse clinical outcome, although this was not clinically significant (p<0.0691). Additionally, two patients who received prior radiosurgery at another institution displayed no adverse effects to retreatment.

The optimal dose for treatment of TN has varied in the literature. It has been suggested that doses of more than 70 Gy have better sustained pain relief than those treated with less than 70 Gy using Gamma Knife [7,16]. Radiation doses of 80-90 Gy have been used with similar results [15,23-24]. However, Pollock, et al. demonstrated statistically higher permanent facial trigeminal nerve dysfunction (54%) and higher dysthesias (32%) after 90 Gy vs. 70 Gy dosage using Gamma Knife [23]. Other reports of post-treatment sensory changes range from 6%-54% [7,16,23]. Villavicencio, et al. reported on a series of 95 patients who used CyberKnife for the treatment of TN and noted that facial numbness was more likely to occur at higher doses but were predictive of a lower recurrence rate [21]. Similarly, higher doses using LINAC-based systems were associated with high rates of trigeminal nerve dysfunction [16,22-23]. In a series of 20 patients undergoing frame-based LINAC radiosurgery, Zahra et al. gave a dose of 80-90 Gy to a single isocenter and advocated to change the isodose line touching the brainstem to 40% of the treatment dose to limit toxicity accounting for the larger collimator used [10]. Since then, published data have employed doses ranging from 80-100 Gy [22,25-26]. In our experience, we did not see an increase in brainstem toxicity when using doses of 90 Gy compared to published data with lower prescriptions. The maximum dose prescription should be left to the comfort of the provider. However, we advocated the use of 90 Gy to a single isocenter with the 50% isodose line abutting the brainstem. Our results did not show any signs of permanent brainstem toxicity.

Pain relief latency also varies depending on the dose and system used. Time to pain relief remains shortest using CyberKnife, with reports of pain relief in as little as 7-14 days to 2 months [8,21]. This is much shorter than the reported times using Gamma Knife or other LINAC-based systems [4,7,16,22-23]. Our experience is similar to other published LINAC-based studies because patients took an average of 82 days (median 42 days; range 7-365 days) to experience complete pain relief.

Pain relief and the rates of facial numbness in both CyberKnife and LINAC-based systems are comparable to Gamma Knife radiosurgery. Patients undergoing CyberKnife have excellent pain relief of 50%-72% for two years following treatment [21,27-28]. Frame-based LINAC systems have also reported pain relief between 76% and 95% over a follow-up of 21 months [22,26]. Smith et al. initially reported on a series of 60 patients in 2003, followed by a second series of 179 patients in 2011; they used LINAC for TN and found a dose-dependent response to pain control with 90 Gy to the 50% IDL achieving pain relief in 88% of the patients [29]. In the only entire frameless LINAC study published, Chen et al. reported a series of 44 patients with a 15-month follow-up. They found an excellent (BNI PS I and II) pain relief of 52% and a recurrence rate of 25% [9]. In our study, 61% of patients had excellent (BNI PS I only) pain relief and 72% of patients had successful outcomes after a follow-up of 27.5 months—the longest frameless LINAC patient series to date. Although patients with TN type 2, a history of multiple sclerosis, prior surgical intervention, and higher BNI PS had lower pain-free survival, reports of pain improvement are comparable to both Gamma Knife and frame-based LINAC systems. In addition, three of the five patients (60%) with prior surgery or radiosurgery treatment had successful results after treatment at our institution. This remains similar to other frame-based treatment modalities and is higher than that reported by Chen et al., who reported 45% successful retreatment [9].

Facial numbness remains the most common complication after radiosurgery to treat TN. Facial numbness has been reported in 23.8%-35% which has been correlated to prior surgery and a larger collimator [10,26]. In one of the largest series that documents the use of LINAC radiosurgery for the treatment of TN, Smith et al. reported that 49% of patients experienced ipsilateral facial numbness [29]. Frameless hypofractionated radiosurgery has been used with similar findings [30]. In the only LINAC frameless study published, Chen, et al. reported 11% having facial numbness and a recurrence rate of 25% [9]. However, recurrence rates as high as 63% have been reported when using a LINAC-based system [25]. In our study, 39% of patients experienced facial numbness but recurrence was only noted in 17%. We failed to show statistical significance in the rate of facial numbness in patients with a prior history of surgery, although 60% of those who had a prior operation experienced facial numbness versus 36% of those who did not have prior surgery (p<0.6673).

In our study, we found successful pain relief in 72% of patients after a median follow- up of 28.5 months, similar to the reports of other LINAC or CyberKnife studies. Post-treatment MRIs revealed enhancement at the root entry zone in six patients, five of which had excellent results; yet, this was not a statistically significant (p<1.0) predictor of treatment success.

The limitations of this study include the difficulty in evaluating and comparing radiosurgery literature, as each published report uses different outcome measures for treatment success, different dosing strategies, and different ways of defining complications. Although attempts at standardizing patient outcomes have made great progress, a uniform method of measuring success has yet to be determined. This is evident when looking at the Chen et al. series, where excellent results were defined as BNI PS I and II [9]. Another limitation includes the small number of patients. There are only a few patient series reporting frameless radiosurgery techniques for the treatment of TN. A follow-up of over two years has yet to be reported. This is needed to add valuable data to the growing literature on radiosurgery treatment for TN. However, the need for a multicenter randomized study and larger patient cohort is needed to draw any statistical conclusions.

Although Gamma Knife is considered the gold standard radiosurgery treatment for TN, the Truebeam system provides a frameless alternative. The safety and efficacy established in this analysis demonstrates an important opportunity for centers to provide effective treatment of TN in the absence of a Gamma Knife platform. The novelty of this system is that it is a frameless system, and it adds to the body of the literature.

## Conclusions

TN can be effectively and safely treated using the Varian Truebeam system, with results similar to that published in the literature. Although statistically significant findings were not present, patients with TN type 1 tend to recover better than those with other forms of TN; they also experience longer pain-free survival. In addition, patients with prior surgical or radiosurgical treatment may benefit from further radiation with the Truebeam system. Patients with prior radiation treatment did not have an increased incidence of facial numbness. The follow-up results of over two years are promising for the continued success of the Truebeam system for the treatment of TN.
